# Measuring Stereotype Threat at Math and Language Arts in Secondary School: Validation of a Questionnaire

**DOI:** 10.3389/fpsyg.2021.553964

**Published:** 2021-06-28

**Authors:** Sylwia Bedyńska, Piotr Rycielski, Magdalena Jabłońska

**Affiliations:** ^1^Center for Research on Social Relations, Institute of Psychology, SWPS University of Social Sciences and Humanities, Warsaw, Poland; ^2^Institute of Psychology, SWPS University of Social Sciences and Humanities, Warsaw, Poland

**Keywords:** stereotype threat, mathematical achievement, language achievement, measurement invariance, confirmatory factor analysis

## Abstract

A stereotype threat arises when a negative stereotype about group to which an individual belongs is activated. It affects the achievement and interest of students in a particular academic domain, e.g., girls at math or boys at language arts. Hence, it is important to assess the level of stereotype threat at school (STaS) in order to identify the vulnerability of students to its negative consequences. This study devised and validated two parallel versions of the STaS scale: girls in mathematics and boys in language arts in a nationally representative sample of Polish secondary school students (*N* = 1,241; 13–16 years). The results of a confirmatory factor analysis (CFA) in a complex sample approach showed one general factor. Furthermore, a multiple-group CFA confirmed metric invariance and partial scalar invariance. The variances for boys and girls were equal. This suggests that the construct of stereotype threat is similarly conceptualized by both genders despite being in different domains. Finally, the comparison of means of latent variables revealed a higher level of stereotype threat among boys in the language domain than girls in mathematics. Possible theoretical and practical implications are discussed.

## Introduction

The causes of gender differences in mathematics have been extensively examined over the years of research (refer meta-analysis: Else-Quest et al., 2010). However, although the gender gap in mathematics favoring boys is still substantial in some domains (e.g., geometry), recent data revealed a much wider gap in reading in favor of girls (Stoet and Geary, 2013; Moè et al., 2021). For example, data collected by the Programme for International Student Assessment (PISA) in 75 countries confirmed that gender differences in reading were three times higher than those in mathematics. Moreover, the gender gap in reading, in contrast to the one in mathematics, has been widening in the following years according to the assessment conducted by the PISA. This trend identified from the PISA research suggests that there is a need for a better understanding and an adequate explanation for gender differences in distinct academic domains, e.g., in STEM (Science, Technology, Engineering, and Mathematics), men outperform women, and in social sciences and humanities, the pattern is reversed.

### Stereotype Threat

Several psychological factors were proposed to explain gender differences in mathematics and language (Ceci et al., 2009; Galdi et al., 2014; Wang and Degol, 2017; Moè et al., 2021). One of these factors is stereotypical beliefs that are significantly related to mathematical achievement by girls (Dweck, 1999; Moè, 2009, 2018), their interest in math and STEM careers (Garriott et al., 2017), and the number of math courses taken in high school (Starr and Simpkins, 2021). The mechanism underlying the link between gender stereotypes and mathematical performance can be explained by the Stereotype Threat model. According to this model, formulated by Steele and Aronson (1995), when a negative stereotype about a group is cognitively activated in a testing situation, it substantially decreases the achievement of students (for review: Schmader et al., 2008; Pennington and Heim, 2016). For example, when a negative stereotype about women as being worse at math was activated, female students answered correctly fewer items of the mathematical section of Graduate Record Examination (GRE) in comparison to the control condition (Spencer et al., 1999; Schmader, 2002; Cadinu et al., 2005). Not only the standardized tests but also real-life mathematical tasks, such as modular arithmetic, that require a long sequence of subtraction and divisions of two-digit numbers may be affected by the activation of a negative stereotype (Beilock and Carr, 2005; Beilock et al., 2007). Considering that this pattern of results was corroborated in numerous studies, several meta-analyses have been published to date to examine the size of stereotype threat effects for females in mathematics and its potential moderators (Nguyen and Ryan, 2008; Doyle and Voyer, 2016). In the most recent meta-analysis, Doyle and Voyer (2016) found a significant effect of stereotype threat on the performance of women using a sample of 133 relevant articles, with a diverse sample of participants comprising participants of different ages and nationalities and a variety of mathematical tasks used as dependent variables. Primarily, although the mean effect size of stereotype threat manipulations was rather small (*d* = 0.29), it significantly differed from zero, confirming the effect of stereotype threat on mathematical performance.

To explain the underperformance in stereotype threat situations, an integrated process model (Schmader et al., 2008) that describes the cognitive and affective processes linking stereotype threat and performance deficits observed in many stereotyped groups was proposed. Among the central processes initiated by a negative stereotype, the model identifies physiological stress response, high vigilance, appraisal processes, suppression of negative thoughts, negative emotions, and limitations of executive functions of working memory. The research on the effects of stereotype threat in mathematics has also confirmed the mediational role of these factors in decreasing the performance of female participants in mathematical tasks (Bosson et al., 2004; Cadinu et al., 2005). As established in some studies, negative stereotype activation affects performance by increasing anxiety (Bosson et al., 2004) and evoking negative and intrusive thoughts (Cadinu et al., 2005). In consequence, attempts to self-regulate these thoughts and emotions under stereotype threat create a high load on working memory resources (Schmader and Johns, 2003), specifically on the phonological loop, in which a verbal material, such as intrusive thoughts, is processed (Beilock and Carr, 2005; Beilock et al., 2007). It was also demonstrated that the limited working memory capacity not only undermines the performance in mathematical tasks in female samples but also can affect tasks that are unrelated to the activated stereotype when depending on working memory resources. These results demonstrated that stereotype threat effects may spill over into other domains unrelated to the stereotype in question, but highly related to the resources of working memory.

The importance of considering stereotype threat in studies on student performance is evident for female samples and mathematics. Recent studies indicate, however, that boys score lower than girls in reading, yet the role of stereotype threat has rarely been studies in a male sample, with only few studies investigating stereotype thread in reading (Pansu et al., 2016). To fill this gap, in previous studies, Bedyńska et al. (2020) examined the association of the level of the stereotype threat and school achievement in two groups: boys in language arts and girls in mathematics (Bedyńska et al., 2018). We presented significant associations between the level of stereotype threat and mathematical achievement in the sample of girls (Bedyńska et al., 2018). Analogously, a similar pattern of results was obtained in the sample of boys in language arts; a higher level of stereotype threat experienced by boys in language arts was associated with lower achievement in this domain (Bedyńska et al., 2020). Given the results of this line of research, several new questions may be posed. First, the question arises whether the level of stereotype threat may be compared across gender groups when measured with regard to different domains: mathematics and language arts. In such a case, can we observe a similar pattern as in the PISA studies, with a higher level of stereotype threat in boys in language arts than in girls in mathematics? Second, it is interesting to investigate whether the mechanism of stereotype threat is similar across gender (for review: Pennington and Heim, 2016). Without confirming the psychometric validity of the Stereotype Threat at School (STaS) scale and measurement equivalence of its parallel forms, boys in language arts and girls in mathematics, these research problems may not be reliably examined. In the present study, we address these issues to open the venue for further investigation.

#### Stereotype Threat Measures

A variety of measures have been proposed in the literature to evaluate the level of stereotype threat in occupational and educational settings (for review: Woodcock et al., 2012; Xavier et al., 2014; Deemer et al., 2016). One of the first assessments of stereotype threat was proposed by Steele and Aronson (1995). The study items described worries of Afro-American students when solving a verbal test or at English lessons, for example “Some people feel I have less verbal ability because of my race.” Worries of being stereotypically perceived by others and negative thoughts distracting attention from the task at hand due to the activation of stereotype content have been also used as measures of core symptoms of stereotype threat. Mostly, they have been employed in two domains, at work, samples of female (von Hippel et al., 2011, 2017; Bedyńska and Zołnierczyk-Zreda, 2015; Manzi et al., 2019), male (Kalokerinos et al., 2017), and older employees (von Hippel et al., 2013, 2019), and in education, in Afro-American and Latino samples (Woodcock et al., 2012).

Although unidimensional self-report scales, inspired by the one used in the classical work of Steele and Aronson (1995), are the most widespread, other measures were also proposed (Kiefer and Sekaquaptewa, 2007; Pseekos et al., 2008; Moè, 2009; Galdi et al., 2014; Deemer et al., 2016). For instance, the Academic Stereotype Threat Inventory (ASTI, Pseekos et al., 2008) consists of three subscales, with items assessing the following: (1) worries of being perceived stereotypically, (2) stereotype endorsement and awareness, and (3) math interest and abilities. Some authors also proposed tools assessing stereotype threat with the use of implicit measures (Kiefer and Sekaquaptewa, 2007; Moè, 2009; Galdi et al., 2014) aiming to evaluate automatic associations consistent with a particular stereotype that may evoke stereotype threat. Although implicit measures of stereotype threat proved to be theoretically valid in predicting underperformance in a stereotyped domain (especially in young children) (Moè, 2009; Galdi et al., 2014), their mode of administration, requiring precise measurement of reaction times, blocks its usage in practice as a screening tool to identify students vulnerability to stereotype threat performance deficits. Similar obstacles are related to multidimensional scales. Therefore, self-report measures seem to be more suitable for this purpose.

Overall, the review of the literature indicates that a vast majority of existing measures of stereotype threat are unidimensional. The external validity of short unidimensional scales of stereotype threat is well-documented in different domains: in occupational settings (von Hippel et al., 2011, 2013, 2017, 2019; Bedyńska and Zołnierczyk-Zreda, 2015; Kalokerinos et al., 2017; Manzi et al., 2019) and educational settings (Woodcock et al., 2012; Bedyńska et al., 2018, 2019, 2020). In the context of academic learning, Woodcock and colleagues (Woodcock et al., 2012) used a short scale to measure worries of the participants to be perceived by others based on their ethnicity. The results of the psychometric analysis confirmed the assumed unidimensionality of the scale and its measurement invariance across both ethnic groups. More importantly, in longitudinal studies, researchers demonstrated the relation of stereotype threat with academic identification and the intention to pursue an academic career in Latino and African American students. In previous investigations by Bedyńska et al., we examined the relationship between stereotype threat and school achievement in two groups: girls in mathematics (Bedyńska et al., 2018) and boys in language arts (Bedyńska et al., 2020). We used a self-descriptive measure of stereotype threat (STaS scale) to assess worries of girls and boys, confirming negative stereotypes in particular subjects. We demonstrated a significant link between the level of stereotype threat, working memory capacity, and mathematical achievement in the sample of girls (Bedyńska et al., 2018). The same associations were obtained in the sample of boys with a higher stereotype threat in language arts being related not only to lower working memory capacity and lower achievement in language arts but also to domain disidentification (Bedyńska et al., 2020). Similar to the study of Woodcock et al. (2012), these results provided preliminary support for the predictive validity of the self-report scales measuring stereotype threat.

### The Aim of the Current Study

Following the work of Woodcock et al. (2012), we believe that the scale that allows the comparison of the level of stereotype threat among different gender groups and different domains is of great practical and theoretical importance. In the real world, group diversity considerably grows, also in educational settings (Junn, 2004; Rougier and Honohan, 2015). As a result, one class may gather students who belong to different stereotyped groups due to gender, race, or ethnic groups, to name a few. A measure comparably assessing stereotype threat across groups and domains of knowledge may be a prominent tool in identifying the most vulnerable students who may benefit most from interventions reducing stereotype threat. For the same reason, proposing a self-descriptive tool for younger participants seems essential as stereotype threat may shape their choices of a study major at educational stages earlier than at the university level. We suggest that such scales offer important benefits to the field of studies on stereotype threat by providing further insight into whether the underlying mechanism of observed stereotype threat effects is the same for different groups, different stereotypes, and different domains. Therefore, validating such a tool may be an important step in a theoretical inquiry, regardless of whether there is one or many stereotype threats as discussed by Shapiro and Neuberg (2007).

The purpose of this study is a psychometric validation of two parallel forms of the STaS scale: girls in mathematics and boys in language arts. Similar to previous scales, we designed items based on Steele and Aronson (1995) that refer to two different sources of stereotype threat: teachers and colleagues. The decision of authors was driven by experimental research on stereotype threat that collected numerous situational triggers of stereotype threat (for review: Schmader et al., 2008), with such subtle ones as the limited number of students belonging to stereotyped groups at class (Inzlicht and Ben-Zeev, 2000; Pennington and Heim, 2016) or the experimenter group membership opposite to that of a subject (Stone and McWhinnie, 2008). In this study, we present data confirming the reliability and construct validity of the STaS scale in a representative sample of students. We assume that the stereotype threat measure should be unidimensional and we test this assumption using Confirmatory Factor Analysis (CFA). However, given some theoretical considerations on different mechanisms of stereotype threat depending on the source of that threat (Stone and McWhinnie, 2008), we also test two alternative models in a CFA: a two-factor model (general stereotype threat and stereotype threat in relations to others) and a three-factor model (general stereotype threat, stereotype threat in relation with a teacher, and stereotype threat in relations with colleagues).

Second, the aim of this study is not only to present empirical data confirming the reliability and construct validity of the STaS scale but also to confirm the psychometric equivalence of the proposed parallel forms of the scale: girls in mathematics and boys in language arts. Therefore, we evaluate measurement invariance of the STaS scale across gender in two different contexts, mathematics and language arts, applying a widely used Multi-Group CFA method. In metric invariance, we test whether the pattern and values of factor loadings are equal across gender groups. Invariance, in that aspect, means that a measurement bias with respect to groups is absent and the construct is similarly conceptualized by participants in both gender groups. Therefore, we examine scalar invariance to confirm that item intercepts are equal. Scalar invariance is important to compare latent means, and, when confirmed, it informs that participants with the same score on the STaS scale have the same level of stereotype threat. We also test two aspects of structural invariance: factor variance and factor mean invariance to examine if the variances and means of the latent construct differ across gender groups. This study aims to investigate whether girls and boys have equivalent amounts of individual differences in stereotype threat (variance equivalence) and whether the average level of stereotype threat is different for girls and boys.

## Method

### Participants and Procedure

The final sample consisted of 619 male secondary school students (13–16 years, *M*_*age*_ = 14.53 years, *SD* = 0.91), with 231 boys at the first grade level (*M*_*age*_ = 13.55 years, *SD* = 0.42), 204 at the second grade level (*M*_*age*_ = 14.5 years, *SD* = 0.37), and 184 in the third grade level (*M*_*age*_ = 15.54 years, *SD* = 0.38), and 622 female secondary school students with a mean age of 15 years old (*M*_*age*_ = 14.59 years, *SD* = 0.92). There were 214 girls in the first grade level (*M*_*age*_ = 13.52 years, *SD* = 0.31), 195 in the second grade level (*M*_*age*_ = 14.62 years, *SD* = 0.52), and 213 in the third grade level (*M*_*age*_ = 15.54 years, *SD* = 0.31).

The sampling procedure had two steps. In the first step, 24 secondary schools were randomly sampled from SIO (*System Informacji Oświatowej*—*Eng. Polish School Database System*) with the stratification based on the voivodeships of Poland (Masovian Voivodeship and Lublin Voivodeship) and the size of the city (45% of schools were selected from big cities and 55% of schools were chosen from small towns and villages). The sampling frame did not include schools for adults, special schools, schools by hospitals or prisons, and the very small ones, with <10 students in a class. In the second step, in each school, two to five classes were randomly selected. The present study was conducted in compliance with the ethical standards adopted by the American Psychological Association (American Psychological Association, 2017). Accordingly, prior to participation, students were informed about the general aim of the research and the anonymity of their data. The participation was voluntary, and the students did not receive compensation for their participation in the study. Additionally, parents signed a written consent form for their children to participate in the study. Data were collected in a single session using an online platform and an online questionnaire, which the students filled in during regular school hours.

### Measures

#### Stereotype Threat at School Scale

Chronic stereotype threat was assessed using two parallel versions of the STaS scale. Given that negative stereotypes about boys and girls describe both groups as particularly weak at different subjects, we constructed two sets of items: the first for girls describing stereotype threat during mathematics lessons and the second for boys describing stereotype threat at language arts lessons. In each version, we used seven items from previous works of Bedyńska et al. (2018, 2019, 2020). To cover different sources of stereotype threat identified in the literature (Stone and McWhinnie, 2008), we proposed two items for measuring specific stereotype threat of being judged by other students (e.g., “I am afraid that some of my friends think that I have much lower math skills because I am a girl”), two items of being judged by teachers (e.g., “I am afraid that my math teacher will think that I may not succeed because I am a girl”), and three items for measuring generalized stereotype threat (e.g., “When I take a math test I am afraid that my low score will confirm a stereotype that girls have lower math skills”). Participants answered using a 6-point scale, ranging from 1 (*strongly disagree*) to 6 (*strongly agree*). All items in the native language as well as their English translation are presented in Table 1.

**Table 1:** Items of two versions of the Stereotype Threat at School (STaS) Scale: girls in mathematics and boys in language arts in the original version and English translation.

**Polish version**	**English translation**
**STaS VERSION FOR GIRLS**	
Item 1. Boję się, że niektórzy moi koledzy i kolezanki myślą, że mam zdecydowanie mniejsze zdolności matematyczne ponieważ jestem dziewczyną.	Item 1. I am afraid that some of my friends think that I have much lower math skills because I am a girl.
Item 2. Gdy piszę sprawdzian z matematyki niepokoję się, że mój słaby wynik potwierdzi stereotyp, że dziewczyny nie mają zdolności matematycznych.	Item 2. When I take a math test I am afraid that my low score will confirm a stereotype that girls have lower math skills.
Item 3. Niektórzy moi koledzy i koleżanki sądzą, że dziewczyny nie powinny się zajmować przedmiotami ścisłymi, bo to mało kobiece.	Item 3. Some of my friends think that girls should not be interested in science because it is not feminine.
Item 4. Czasem przychodzi mi do głowy, że przecież nie powinnam być dobra z matematyki, bo jestem dziewczyną.	Item 4. Sometimes I think that I should not be good in math because I am a girl.
Item 5. Obawiam się, że mój nauczyciel matematyki może uznać, że sobie nie poradze poniewaz jestem dziewczyną.	Item 5. I am afraid that my math teacher will think that I may not succeed because I am a girl.
Item 6. Na lekcjach matematyki czuję niepokój, że jeśli gorzej mi pójdzie to będzie to potwierdzenie tego, że dziewczyny nie powinny się zajmować się matematyką.	Item 6. During math classes I feel anxious that if I do not succeed I will reinforce a stereotype that girls should not be interested in math.
Item 7. Czasami przychodzi mi do głowy, że na matematyce jestem oceniana przez pryzmat płci a nie rzeczywistych zdolności.	Item 7. I sometimes think that when it comes to math I am evaluated based on my gender and not my actual abilities.
**STaS VERSION FOR BOYS**	
Item 1. Boję się, że niektórzy moi koledzy i koleżanki myślą, że mam zdecydowanie mniejsze zdolności językowe poniewaz jestem chłopcem.	Item 1. I am afraid that some of my friends think that I have much lower language skills because I am a boy.
Item 2. Gdy piszę sprawdzian z języka polskiego niepokoję się, że mój słaby wynik potwierdzi stereotyp, że chłopcy nie mają zdolności językowych.	Item 2. When I take a language test I am afraid that my low score will confirm a stereotype that boys have lower language skills.
Item 3. Niektórzy moi koledzy i kolezanki sądzą, że chłopcy nie powinni się zajmować przedmiotami humanistycznymi, bo to mało męskie.	Item 3. Some of my friends think that boys should not be interested in language arts because it is not masculine.
Item 4. Czasem przychodzi mi do głowy, że przeciez nie powinienem być dobry z jezyka polskiego, bo jęstem chłopcem.	Item 4. Sometimes I think that I should not be good in language because I am a boy.
Item 5. Obawiam się, że mój nauczyciel języka polskiego może uznać, że sobie nie poradzę ponieważ jestem chłopcem.	Item 5. I am afraid that my language teacher will think that I may not succeed because I am a boy.
Item 6. Na lekcjach języka polskiego czuję niepokój, że jeśli gorzej mi pójdzie to będzie to potwierdzenie tego, że chłopcy nie powinni się zajmować się literaturą.	Item 6. During language classes I feel anxious that if I do not succeed I will reinforce a stereotype that boys should not be interested in language arts.
Item 7. Czasami przychodzi mi do głowy, że na języku polskim jestem oceniany przez pryzmat płci a nie rzeczywistych zdolności.	Item 7. I sometimes think that when it comes to language I am evaluated based on my gender and not my actual abilities

### Data Preparation and Analytical Approach

All analyses were conducted using Mplus 7.3 (Muthén and Muthén, 1998–2015). We used structural equation modeling with complex sampling (analysis type: complex) and Maximum Likelihood Robust (MLR) approach implemented into Mplus to deal with clustered data (students nested in classes) and a model that contains continuous non-normal distributed variables (Muthén and Satorra, 1995). In the first step, all classes smaller than three students were excluded from the analysis (seven classes, 15 participants). First, an exploratory analysis of the data was performed with descriptive statistics and correlations to evaluate the quality of the data. Then, a CFA was conducted. We specified three models with one, two, or three factors loaded by seven STaS scale items. In the two-factor model, we assumed that three items were loaded onto general stereotype threat and four items were loaded onto stereotype threat in relation to others: other students and teachers. In the three-factor model, we also separated the latter source of stereotype threat into two components: stereotype threat in relation to colleagues and stereotype threat in relation to teachers. All CFA models were evaluated using fit indices following the recommendations of Kline (2011). We used Root Mean Square Error Approximation (RMSEA), Standardized Root Mean Square Residual (SRMR), the Comparative Fit Index (CFI), and the Tucker-Lewis Index (TLI), as well as the general fit based on X^2^ test of model fit and its significance (*p*). We adopted the most widely recommended cut-off values indicative of an adequate model fit to the data: RMSEA and SRMR <0.06 and <0.08, CFI and TLI >0.95 and >0.90, respectively (Lance et al., 2006).

The analysis of multiple-group invariance was conducted to determine the extent to which the factor structure was comparable across gender. As suggested in the literature (e.g., Milfont and Fisher, 2010), five aspects of measurement invariance (configural, metric, scalar, residual variance, and residual covariance) and two aspects of structural invariance (factor variance and factor mean) were tested with different sets of equality constraints on model parameters. Given the well-known sensitivity of the chi-square test of model fit to sample size (Bentler and Bonett, 1980), the comparison of models was based not only on the overall model fit differences but also on the change of CFI (Chen, 2007) and RMSEA values (Rutkowski and Svetina, 2014). In particular, a change in CFI equal to or greater than 0.010, supplemented by a change in RMSEA ≤ 0.015 were considered as indicators of measurement invariance (Chen, 2007).

## Results

### Descriptive Statistics for Items

Descriptive statistics for all items and the parallel version of the STaS scale in two gender groups are presented in Table 2. The index for stereotype threat was calculated as the mean of the answers of a participant to all items of the STaS scale. Generally, means were quite low but the SDs were acceptable. An inspection of the distribution statistics shows that there was no high asymmetry of empirical distributions of the responses. All item-total correlations were high with values above 0.85 in both samples. Table 3 reports Spearman's rho coefficients across items for girls and boys showing moderate and high correlations between items in both groups.

**Table 2:** Descriptive statistics of the STaS items and the STaS scale in gender groups.

**Statistics**	**Item 1**	**Item 2**	**Item 3**	**Item 4**	**Item 5**	**Item 6**	**Item 7**	**STaS Scale**
**GIRLS**								
Mean	2.21	2.13	2.07	1.96	1.88	1.86	1.79	1.99
Standard deviation	1.43	1.44	1.46	1.45	1.39	1.36	1.32	1.08
Skewness	1.19	1.19	1.40	1.50	1.65	1.68	1.80	1.32
Kurtosis	0.67	0.52	1.03	1.29	1.83	1.96	2.56	1.74
Item-total correlation	0.87	0.86	0.88	0.87	0.86	0.86	0.87	–
**BOYS**								
Mean	2.12	2.29	2.44	2.35	2.36	2.24	2.39	2.31
Standard deviation	1.43	1.49	1.49	1.51	1.61	1.54	1.59	1.16
Skewness	1.25	1.01	0.83	0.90	1.00	1.11	0.95	0.81
Kurtosis	0.78	0.10	−0.12	−0.17	−0.13	0.23	−0.21	0.26
Item-total correlation	0.88	0.86	0.87	0.87	0.87	0.87	0.87	–

*An index for the level of stereotype threat was calculated as the mean of the answers of a participant to all items of the STaS scale*.

**Table 3:** Correlation coefficients for gender groups.

**Spearman's rho for girls (right upper half of the matrix) and boys (left lower half of the matrix)**							
	**Item 1**	**Item 2**	**Item 3**	**Item 4**	**Item 5**	**Item 6**	**Item 7**
Item 1	–	0.64^**^	0.50^**^	0.44^**^	0.50^**^	0.53^**^	0.40^**^
Item 2	0.68^**^	–	0.53^**^	0.57^**^	0.59^**^	0.61^**^	0.49^**^
Item 3	0.55^**^	0.63^**^	–	0.51^**^	0.46^**^	0.49^**^	0.43^**^
Item 4	0.49^**^	0.60^**^	0.60^**^	–	0.62^**^	0.62^**^	0.55^**^
Item 5	0.49^**^	0.58^**^	0.50^**^	0.59^**^	–	0.72^**^	0.67^**^
Item 6	0.46^**^	0.58^**^	0.55^**^	0.60^**^	0.66^**^	–	0.69^**^
Item 7	0.41^**^	0.55^**^	0.46^**^	0.54^**^	0.68^**^	0.61^**^	–

** *Correlation significant at p = 0.01 level*.

### CFA of the STaS Scale

Construct validity was tested using CFA separately in both gender groups. Following theoretical assumptions, three models were tested with one, two, and three latent factors. In the one-factor solution, we assumed that the scale was unidimensional and all items reflected stereotype threat. In the two-factor model, item 1, item 3, item 5, and item 7 formed the first factor (stereotype threat in relationships with others) and item 2, item 4, and item 6 formed the second factor (general stereotype threat). In the three-factor model, items 1, 3, 5, and 7 were split into two factors, forming, respectively, the second factor, stereotype threat in relationships with colleagues (item 1 and item 3), and the third factor, stereotype threat in relationships with a teacher (item 5 and item 7). Items measuring general stereotype threat remained the same as in the two-factor model. The CFA results for all models are presented in Table 4.

**Table 4:** Model fit statistics for confirmatory factor analysis (CFA) of the STaS scale in gender groups.

**Model**	**X^**2**^**	***df***	**CFI**	**TLI**	**SRMR**	**RMSEA, [90% CI]**
**GIRLS**						
M1—one factor^a^	23.93^*^	11	0.99	0.98	0.02	0.04 [0.02, 0.06]
M2—two factors^b^	93.56^***^	13	0.93	0.89	0.04	0.09^*^ [0.07, 0.10]
M3—three factors^c^	60.11^***^	11	0.96	0.92	0.04	0.07^*^ [0.06, 0.09]
**BOYS**						
M1—one factor^a^	36.86^***^	12	0.98	0.96	0.03	0.06 [0.04, 0.08]
M2—two factors^b^	107.62^***^	13	0.91	0.86	0.05	0.11^**^ [0.09, 0.13]
M3—three factors^c^	45.11^**^	11	0.97	0.94	0.03	0.07 [0.05, 0.09]

*Structural equation modeling with Maximum Likelihood Robust (MLR) estimation was used for the analyses. NFI, normed fit index; CFI, comparative fit index; RMSEA, root-mean-square error of approximation*.

a *In Model M1, all items of stereotype threat were loaded onto one factor*.

b *In Model M2, the three items were loaded onto the general stereotype threat and the four items were loaded into stereotype threat in relation to others (teacher and colleagues)*.

c *In Model M3, the three items were loaded onto general stereotype threat, the two items were loaded onto stereotype threat in relation with teachers, and the two items were loaded onto stereotype threat in relation with colleagues*.

* *p < 0.05*,

** *p < 0.01*,

*** *p < 0.001*.

For girls, the inspection of modification indices in one-factor solution suggested that significant covariance between items 1 and 2 (0.31), items 5 and 7 (0.27), and items 6 and 7 (0.26) should be entered to obtain a good fit of the model. The CFA results for this modified one-factor model with covariances between items achieved a good overall fit. Similar covariance of error terms between some items was also reported by Woodcock et al. (2012). The statistics for the two-factor model and the three-factor model were a bit worse (as shown in Table 4).

For boys, we also added in one-factor solution covariance between items 7 and 5 (0.35) as well as items 1 and 2 (0.34), as suggested by modification indices to achieve a good fit. The model with covariances obtained the good values of specific fit indices. The two-factor model presented a slightly lower general fit. A similar, relatively lower fit was obtained in the three-factor model so that a general fit was lower than that for the unidimensional model with fit indices values presenting a good fit. Factor loadings for all tested models are presented in Table 5. All factor loadings for one-factor model were moderate and high.

**Table 5:** Model factor loadings and residual variances (in parentheses) for gender groups for three tested models.

	**M1—one factor^a^**	**M2—two factors^b^**	**M3—three factors^c^**
**GIRLS**			
Item 1	0.60 (0.48)	0.62 (0.62)	0.69 (0.52)
Item 2	0.72 (0.59)	0.72 (0.49)	0.73 (0.47)
Item 3	0.64 (0.48)	0.62 (0.61)	0.67 (0.55)
Item 4	0.72 (0.40)	0.70 (0.51)	0.70 (0.52)
Item 5	0.78 (0.40)	0.79 (0.38)	0.84 (0.30)
Item 6	0.78 (0.56)	0.80 (0.37)	0.79 (0.38)
Item 7	0.66 (0.64)	0.73 (0.47)	0.78 (0.40)
**BOYS**			
Item 1	0.63 (0.61)	0.67 (0.56)	0.72 (0.49)
Item 2	0.76 (0.42)	0.78 (0.39)	0.79 (0.38)
Item 3	0.70 (0.51)	0.69 (0.53)	0.74 (0.46)
Item 4	0.76 (0.43)	0.73 (0.46)	0.73 (0.46)
Item 5	0.71 (0.49)	0.72 (0.48)	0.85 (0.29)
Item 6	0.75 (0.44)	0.73 (0.47)	0.72 (0.48)
Item 7	0.65 (0.58)	0.66 (0.56)	0.77 (0.41)

*Structural equation modeling with MLR estimation was used for the analysis*.

a *In Model M1, all items of stereotype threat were loaded onto one factor*.

b *In Model M2, the three items were loaded onto the general stereotype threat and the four items were loaded into stereotype threat in relation to others (teacher and colleagues)*.

c *In Model M3, the three items were loaded onto general stereotype threat, the two items were loaded onto stereotype threat in relation with teachers, and the two items were loaded onto stereotype threat in relation with colleagues*.

To summarize, given theoretical assumptions and the results of CFA, a one-factor model with covariances between error terms in pairs of items, items 1 and 2, items 5 and 7, and items 6 and 7 in both gender groups, was accepted for further measurement invariance testing.

### Reliability of the Scale

The reliability of the scale was high in both gender groups: Cronbach's α for girls = 0.89 and for boys, Cronbach's α = 0.88. We also calculated reliability statistics separately for three grade levels in gender groups. The reliability in sample of girls was comparatively smaller in the first grade, Cronbach's α = 0.87, than in the second grade, where Cronbach's α = 0.89, while the sample of girls in third grade had the highest level of reliability statistics, Cronbach's α = 0.90. The same pattern was observed in the sample of boys, with Cronbach's α = 0.86 in the first grade, Cronbach's α = 0.89 in the second grade, and Cronbach's α = 0.90 in the third grade. All reliability coefficients met the requirements described in the literature (Furr, 2011).

We also computed the estimated reliability for a one-factor solution separately for girls and boys using the formula presented by Furr (2011, p. 105). For girls, the estimated reliability value was equal 0.82, while for boys it was equal 0.83, showing good reliability. The values were not substantially lower than the Cronbach's α values presented above, indicating high reliability of both versions of the STaS scale.

### Measurement and Structural Invariance of the STaS Scale Across Gender

Based on the theoretical assumptions, previous research (Woodcock et al., 2012), and the results of CFA analyses, a one-factor model with residual covariance between items 1 and 2, items 5 and 7, and items 6 and 7 was tested in a measurement invariance analysis. In the first step, the configural invariance was tested in both gender groups, simultaneously for the re-specified baseline models. As shown in Table 6, the configural level of measurement invariance was achieved. In the metric invariance model, equality of unstandardized item-factor loadings was examined across groups. The model with constraints also fit well and did not differ significantly from the configural model, confirming metric invariance. Such a result may be interpreted as an indication that the same latent factor was measured in girls and boys as the items were related to the factor equivalently in gender groups. To examine scalar invariance, equality of item intercepts across gender was defined. Although the full scalar invariance model fit relatively well to the data, it was significantly worse than the metric model and modification indices suggested some parameters to be constrained. Accordingly, a partial scalar invariance was thus examined with three intercepts (for item 1, item 2, and item 7) being allowed to differ between groups. The model testing partial scalar invariance did not fit significantly worse than the model testing metric invariance, indicating that partial scalar invariance did hold. Such results can be interpreted as an indication that girls are expected to have different item responses than boys in items 1, 2, and 7 at the same absolute level of stereotype threat. The residual invariance model fit well to the data but differed significantly from the partial scalar model. Therefore, the invariance of residual variances was found to be untenable. The finding suggests that item residuals are not the same across groups. Equality of residual covariance between items 1 and 2, items 5 and 7, and items 6 and 7 was tested across groups and was confirmed, showing that residual covariance between items was the same in both samples.

**Table 6:** Fit statistics for measurement invariance by gender.

**Invariance**	**X^**2**^ (df)**	**CF**	**CFI**	**TLI**	**RMSEA [90% CI]**	***p***	**ΔX^**2**^**	**Δ *df***	***p*_**diff**_**
M1 configural	55.623 (22)^*^	2.005	0.98	0.97	0.049 [0.033, 0.066]	0.493			
M2 metric	60.867 (28)^*^	1.876	0.98	0.97	0.043 [0.028, 0.058]	0.753	5.244	6	0.51
M3 scalar	97.198 (34)^*^	1.728	0.97	0.96	0.055 [0.042, 0.067]	0.261	36.331	6	0.001
M3a partial scalar	64.417 (31)^*^	1.796	0.98	0.98	0.042 [0.027, 0.056)	0.824	3.550	3	0.314
M4 residual variance	87.641 (35)^*^	1.869	0.97	0.97	0.049 [0.036, 0.062]	0.525	23.224	4	0.0001
M5 residual covariance	95.288 (38)^*^	1.909	0.97	0.97	0.049 [0.037, 0.062]	0.524	7.647	3	0.054
M6 factor variance	89.730 (36)^*^	1.861	0.97	0.97	0.049 [0.036, 0.062]	0.535	2.089	1	0.148
M7 factor mean	109.373 (37)^*^	1.836	0.96	0.96	0.056 [0.044, 0.068]	0.196	19.643	1	0.001

* *p < 0.05, CF, Correction Factor; CFI, Comparative Fit Index; TLI, Tucker-Lewis Index; RMSEA, Root Mean Square Error Approximation; CI, Confidence Intervals of RMSEA*.

Additionally, two aspects of structural invariance were tested. First, factor variance invariance was examined. The results supported this type of invariance as the model achieved a good fit and did not differ significantly from the residual covariance model. Thus, girls and boys were shown to have equivalent amounts of individual differences in stereotype threat. However, the criteria of factor mean invariance were not met, with boys having a higher level of stereotype threat in language arts than girls in mathematics. All model fit statistics for the test of invariance are given in Table 5.

## Discussion

In the analysis of the PISA data, Stoet and Geary (2013) investigated the magnitude of the gender gap in mathematics and reading and revealed that the gender gap in reading was three times wider than the one in mathematics. This conclusion, redirecting research focus from girls to boys, would have not been possible without methodological advantages of the PISA, with tests of measurement invariance among many. Given that gender differences in achievement are often explained from the perspective of stereotype threat, the recent developments in the measurement of abilities need to be matched by a cross-domain measurement of stereotype threat. This study addressed that need by using the STaS scale to measure stereotype threat in two groups and domains: girls in mathematics and boys in language arts. These parallel versions of the STaS scale were also validated by measurement invariance analysis to present their equivalence in gender groups.

More specifically, the study aimed to provide the validation of two parallel forms of the STaS scale in the representative sample of secondary school students. Although the STaS scale is much shorter in comparison to some existing measures (Pseekos et al., 2008; Deemer et al., 2016), the results confirmed its good reliability in both samples. As predicted, the results supported a unidimensional structure, as shown by the results of a CFA with the best fit for the model with one general factor. These results are also in line with the study involving Latino and Afro-American students conducted by Woodcock et al. (2012), with a scale created based on previous tools used as a manipulation check in experimental research on stereotype threat.

More importantly, we also tested the measurement and structural invariance of the STaS scale across gender groups. By using a multiple-group CFA, we confirmed partial scalar measurement invariance of the STaS scale. We found that all items were similarly related to the latent factor, and the variability of the latent factor was similar across gender groups. These results can be interpreted as proof that stereotype threat can be conceptualized in a similar manner among girls and boys, i.e., by the same type of worries and thoughts. It can be concluded then that, although the STaS scale measures stereotype threat in different domains in the sample of boys and girls, this assessment reflects the same theoretical construct.

This analysis also showed that full structural invariance was not confirmed, as boys were shown to have a higher level of stereotype threat. Possible reasons for departures from full structural invariance may arise from a significant difference in the level of stereotype threat between boys and girls in different school subjects. This gender gap may be explained in the light of the research on stereotype threat and studies on school achievement in language arts and mathematics. Previous research on stereotype threat suggests that this phenomenon may not be singular. As proposed in a multi-threat framework, different factors may elicit stereotype threat, potentially leading to different consequences (Shapiro, 2011). Such a supposition seems to be more plausible than an assumption of identical triggers and mechanisms of stereotype threat in different negatively stereotyped groups. We also believe that being a member of a group with a history of stigmatization (e.g., girls, or African Americans) or a group without such history (e.g., boys, White men) may be an important factor when predicting stereotype threat. Paradoxically, minority members may have developed more effective ways of coping with stereotype threat, and, therefore, their level of stereotype threat may be lower (Ford et al., 2004; Block et al., 2011). If so, it would explain that the lack of factor means invariance. Therefore, we consider this evidence as an important and relevant finding, opening new inquiries about the mechanisms underlying stereotype threat in different groups and the effectiveness of potential interventions. This result may also evoke more interest in exploring the effects of stereotype threat in groups of boys as such research appears to be highly underrepresented in the present literature (e.g., Pansu et al., 2016).

The publication of a reliable and theoretically valid measure of stereotype threat for secondary school children opens avenues for research regarding the long-term consequences of stereotype threat in school settings, such as domain disidentification and a lack of interest in a stereotyped domain. So far, such studies have not been well-represented in the literature. As shown in previous research studies (2019 and 2020), this direction seems very promising and may bring interesting results about the dynamics of coping with stereotype threat and its long-term consequences in students.

### Study Limitations and Future Research Directions

Although the psychometric properties of the STaS scale are promising on a representative national sample of secondary school pupils, further studies in different populations are necessary to fully validate this measure. The tests of external validity should be also considered as an important aim of further research. It is very interesting to investigate whether this measurement tool may be useful with regard to other age groups, for instance, younger (e.g., primary school pupils) or older samples (e.g., high school students or university students), as well as in subjects other than mathematics and language arts. Additionally, further studies are required to evaluate the test–retest stability of stereotype threat evaluations using the STaS scale. Finally, we point out that the validation of this measure opens up avenues for research that would provide an evaluation of practical interventions that can reduce stereotype threat.

### Practical Implications

The presented measurement tool may be also used by practitioners, such as teachers or school psychologists, to select students who should participate in intervention programs designed to reduce the detrimental effects of stereotype threat on performance. Namely, we found that the construct has a similar size of individual differences in both groups, and it provides a reliable assessment of the level of stereotype threat. Using two forms of the STaS scale, pertaining to mathematics and language arts, a teacher can easily identify those pupils of both genders who could benefit from intervention programs reducing stereotype threat. For instance, the new measure can be used to evaluate the effectiveness of different techniques used in a cognitive and behavioral therapy, as it was postulated in a recent work bridging two areas of research, stereotypes and depression (Cox et al., 2012).

## Data Availability Statement

The raw data supporting the conclusions of this article will be made available by the authors, without undue reservation.

## Ethics Statement

The studies involving human participants were reviewed and approved by Ethical Committee of the Educational Research Institute (Warsaw, Poland). Written informed consent to participate in this study was provided by the participants' legal guardian/next of kin.

## Author Contributions

SB made substantial contributions to the conception, design of the work, and acquisition. PR and SB analyzed the data. SB, PR, and MJ interpreted the data for the work, drafted the work, or revised it critically for important intellectual content, approved the final version to be published, and agreed to be accountable for all aspects of the work in ensuring that questions related to the accuracy or integrity of any part of the work are appropriately investigated and resolved. All authors contributed to the article and approved the submitted version.

## Conflict of Interest

The authors declare that the research was conducted in the absence of any commercial or financial relationships that could be construed as a potential conflict of interest.
